# Changes in gut microbiota control inflammation in obese mice through a mechanism involving GLP-2-driven improvement of gut permeability

**DOI:** 10.1136/gut.2008.165886

**Published:** 2009-02-24

**Authors:** P D Cani, S Possemiers, T Van de Wiele, Y Guiot, A Everard, O Rottier, L Geurts, D Naslain, A Neyrinck, D M Lambert, G G Muccioli, N M Delzenne

**Affiliations:** 1 Unit of Pharmacokinetics, Metabolism, Nutrition and Toxicology, Louvain Drug Research Institute, Université catholique de Louvain, Brussels, Belgium; 2 Laboratory of Microbial Ecology and Technology, Faculty of Bioscience Engineering, Ghent University, Gent, Belgium; 3 Department of Pathology, Université catholique de Louvain, Brussels, Belgium; 4 Medicinal Chemistry and Radiopharmacy Unit, Louvain Drug Research Institute, Université catholique de Louvain, Brussels, Belgium; 5 Laboratory of Chemical and Physico-chemical Analysis of Drugs (CHAM), Louvain Drug Research Institute, Université catholique de Louvain, Brussels, Belgium

## Abstract

**Background and aims:**

Obese and diabetic mice display enhanced intestinal permeability and metabolic endotoxaemia that participate in the occurrence of metabolic disorders. Our recent data support the idea that a selective increase of *Bifidobacterium* spp. reduces the impact of high-fat diet-induced metabolic endotoxaemia and inflammatory disorders. Here, we hypothesised that prebiotic modulation of gut microbiota lowers intestinal permeability, by a mechanism involving glucagon-like peptide-2 (GLP-2) thereby improving inflammation and metabolic disorders during obesity and diabetes.

**Methods:**

Study 1: *ob/ob* mice (Ob-CT) were treated with either prebiotic (Ob-Pre) or non-prebiotic carbohydrates as control (Ob-Cell). Study 2: Ob-CT and Ob-Pre mice were treated with GLP-2 antagonist or saline. Study 3: Ob-CT mice were treated with a GLP-2 agonist or saline. We assessed changes in the gut microbiota, intestinal permeability, gut peptides, intestinal epithelial tight-junction proteins ZO-1 and occludin (qPCR and immunohistochemistry), hepatic and systemic inflammation.

**Results:**

Prebiotic-treated mice exhibited a lower plasma lipopolysaccharide (LPS) and cytokines, and a decreased hepatic expression of inflammatory and oxidative stress markers. This decreased inflammatory tone was associated with a lower intestinal permeability and improved tight-junction integrity compared to controls. Prebiotic increased the endogenous intestinotrophic proglucagon-derived peptide (GLP-2) production whereas the GLP-2 antagonist abolished most of the prebiotic effects. Finally, pharmacological GLP-2 treatment decreased gut permeability, systemic and hepatic inflammatory phenotype associated with obesity to a similar extent as that observed following prebiotic-induced changes in gut microbiota.

**Conclusion:**

We found that a selective gut microbiota change controls and increases endogenous GLP-2 production, and consequently improves gut barrier functions by a GLP-2-dependent mechanism, contributing to the improvement of gut barrier functions during obesity and diabetes.

Obesity is typically associated with a cluster of several metabolic disorders, characterised by a low-grade inflammation.^1^ ^2^ Evidence that the gut microbiota composition can be different between healthy and obese and/or type 2 diabetic patients has led to the investigation of this environmental element as a key factor in the pathophysiology of metabolic diseases.^3^^–^5

We previously reported that the gut microbiota is involved in high-fat diet-induced metabolic endotoxaemia, adipose tissue inflammation and metabolic disorders.^6^^–^9 The link between high-fat diet-induced inflammation, oxidative stress, metabolic disorders, and gut microbiota, could be lipopolysaccharide (LPS)-dependent.^7^ ^10^^–^14 Several pieces of clinical and experimental data have confirmed that LPS significantly contributes to the development of obesity-related inflammatory liver diseases such as non-alcoholic fatty liver disease and non-alcoholic steatohepatitis.^15^^–^17 High plasma LPS levels could result from an increased production of endotoxin upon changes in the gut microbiota.^7^ ^8^ Normally, the intestinal epithelium acts as a continuous barrier to avoid LPS translocation; yet some endogenous or exogenous events may alter this protective function. Among the elements promoting a leaky gut, and thus an increased plasmatic LPS level, are alcohol consumption,^15^ ^18^^–^22 immobilisation stress,^23^ ^24^ and radiation^25^ have been proposed. In addition, we have recently shown that the modulation of gut bacteria following a high-fat diet strongly increases intestinal permeability, by reducing the expression of genes coding for two tight junction proteins ZO-1 and occludin.^6^ We previously reported that gut bacteria are clearly involved in these events since obese and high-fat fed-diabetic mice treated with an antibiotic recovered normal intestinal epithelial integrity.^6^ Also, recent data have shown that obese and diabetic mice display enhanced intestinal permeability, and are characterised by a metabolic endotoxaemia and a low-grade inflammation.^6^ ^7^ ^26^ Furthermore, high-fat feeding changes gut microbiota^6^^–^8 27 towards a decreased number of bifidobacteria,^6^^–^8 a group of bacteria which has been shown to reduce intestinal LPS levels in mice and to improve the mucosal barrier function.^28^^–^32 Besides, we have shown that feeding mice with prebiotics increased the number of intestinal bifidobacteria and reduced the impact of high-fat diet-induced metabolic endotoxaemia and inflammatory disorders.^8^ ^33^

Importantly, however, the mechanisms linking prebiotic-induced changes in gut microbiota, metabolic endotoxaemia and the improvement of obesity-related hepatic and metabolic disorders are still unknown.

By using these experiments, we have tested the hypothesis that the control of gut permeability through the selective modulation of gut microbiota by prebiotics participates in the improvement of metabolic diseases in *ob/ob* mice. Novel mechanisms involving the influence of gut fermentation on specific proglucagon-derived peptides – namely, glucagon-like peptide-2 (GLP-2) – are proposed.

## MATERIALS AND METHODS

### Animals

#### Experiment 1

Six-week-old *ob/ob* (n = 10/group) mice (C57BL/6 background; Jackson Laboratory, Bar Harbor, Maine, USA) were housed in a controlled environment (12 h daylight cycle, lights off at 18.00 hours) in groups of 2 mice/cage, and kept with free access to food and water. The mice were fed a control diet (Ob-CT) (A04, Villemoisson sur Orge, France), or a control diet containing a mix of a fermentable dietary fibre (oligofructose) (Ob-Pre) (Orafti, Tienen, Belgium),^33^ or a diet containing a mix a non-fermentable dietary fibre (microcrystalline cellulose) (Ob-Cell) (Vivapur Microcrystalline cellulose; J. Retten Maier 38 Söhne, Weissenborn, Germany). Dietary fibres were added in a proportion of 9:1 (weight of control diet:weight of fibres).

#### Experiment 2

Six-week-old *ob/ob* (n = 8/group) mice (C57BL/6 background; Jackson Laboratory) were housed in a controlled environment (12 h daylight cycle, lights off at 18.00 hours) in groups of 2 mice/cage, and kept with free access to food and water. To study the significance of GLP-2 in this model, mice were injected subcutaneously twice daily for 4 weeks with 2.5 μg/kg of GLP-2 receptor antagonist GLP-2 (3–33) (Eurogentec, Verviers, Belgium) as described^34^^–^36 or saline. The mice were fed a control diet and injected with saline or GLP-2 antagonist (Ob-CT and Ob-Ant, respectively), or fed the prebiotic diet and injected with saline or GLP-2 antagonist (Ob-Pre and Ob-Pre-Ant, respectively).

#### Experiment 3

Six-week-old *ob/ob* (n = 6/group) mice (C57BL/6 background; Jackson Laboratory) were housed in a controlled environment (12 h daylight cycle, lights off at 1800 hours) in groups of 2 mice/cage, and kept with free access to food and water. The mice were fed the same control diet as described in experiment 1. The mice were separated into two groups and injected subcutaneously twice daily for 12 days with 25 μg GLP-2 (1–33) (Bachem, Bubendorf, Switzerland) (Ob-GLP-2), or saline (Ob-CT). GLP-2 doses were based on previous studies describing the physiological effects (ie, the intestinotrophic properties) of the peptides.^35^ ^37^^–^39

### Tissue sampling

Mice were anaesthetised (ketamine/xylazine, intraperineally, 100 and 10 mg/kg, respectively) after a 5 h period of fasting, and blood samples and tissues were harvested for further analysis. Mice were killed by cervical dislocation. Liver, caecum (full and empty), muscles (*vastus lateralis*), and adipose tissues (epididymal, subcutaneous and visceral) were precisely dissected and weighed. The intestinal segments (jejunum, colon) were immersed in liquid nitrogen, and stored at −80°C, for further analysis.

### Microbial analysis of the caecal content of selected mice

Metagenomic DNA was extracted from the caecal content of randomly selected mice (5/group), using the QIAamp DNA stool mini kit (Qiagen, Venlo, Netherlands) according to the manufacturer’s instructions. Denaturing gradient gel electrophoresis (DGGE) on total bacteria, bifidobacteria and lactobacilli were performed to study the qualitative effect of the treatment on the structure and composition of the intestinal microbial community.^40^ DGGE with a 45–60% denaturant gradient were used to separate the polymerase chain reaction (PCR) products obtained with a nested approach for the 16S rRNA genes of bifidobacteria (primers BIF164f-BIF662r) and lactobacilli (SGLAB0158f-SGLAB0667). The first PCR round was followed by a second amplification with primers 338F-GC and 518R. The latter primers were also used to amplify the 16S rDNA of all bacteria on total extracted DNA. The DGGE patterns obtained were subsequently analysed using the Bionumerics software version 2.0 (Applied Maths, Sint-Martens-Latem, Belgium).^41^ In brief, the calculation of the similarities was based on the Pearson (product–moment) correlation coefficient. Clustering analysis was performed using the unweighted pair group method with arithmetic mean clustering algorithm (UPGMA) to calculate the dendrograms of each DGGE gel and a combination of all gels. The latter was performed on a created composite dataset. Multidimensional scaling (MDS) analysis was used to reduce the different data of the complex DGGE patterns of one sample to one point in a three-dimensional space. MDS was based on the combined information from the distance matrices of each DGGE, obtained using similarity coefficients (Pearson correlation).

Quantitative PCR (qPCR) for total bacteria (using primers PRBA338f and P518r) and specific for bifidobacteria, lactobacilli or the *Eubacterium rectale/Clostridium coccoides* grp. was performed to study the quantitative effect of the treatment on the composition of the intestinal microbial community as reported by Possemiers *et al*.^42^

### Real-time qPCR

Total RNA from tissues was prepared using the TriPure reagent (Roche, Basel, Switzerland) as described.^33^ cDNA was synthesised using a reverse transcription kit (Promega, Madison, Wisconsin, USA) from 1 μg of total RNA. qPCR was performed with a STEP one PLUS instrument and software (Applied Biosystems, Foster City, California, USA), as described.^6^ Primer sequences for the targeted mouse genes are presented in supplemental table 1.

**Table 1:** Prebiotic-associated changes in gut microbiota

Bacterial content	Ob-CT	Ob-Cell	Ob-Pre
Total bacteria	5.74^a^ (2.05)	4.30^b^ (0.38)	8.07^c^ (1.05)
*Bifidobacterium* spp	4.65^a^ (1.58)	3.38^b^ (0.37)	6.35^c^ (1.10)
*Lactobacillus* spp	4.93^a^ (1.76)	3.79^a^ (0.38)	7.16^b^ (1.40)
*Clostridium coccoides–Eubacterium rectale* cluster	3.65^a^ (1.37)	2.72^a^ (0.20)	6.41^b^ (1.27)

Results are given as the log DNA copies/caecal content (SD).

Data are mean with the SD.

Data with different superscript letters are significantly different p*<*0.05, according to the post hoc ANOVA statistical analysis.

CT, Cell and Pre, respectively, refer to the selected *ob*/*ob* mice fed a normal-diet, non-prebiotic control diet or prebiotic diet.

### Immunofluorescence analysis of occludin and ZO-1

Jejunum segments were immediately removed, washed with PBS, mounted in embedding medium (Tissue-Tek, Sakura, Netherlands), and stored at −80°C until use. Cryosections (5 μm) were fixed in acetone at −20°C for 5 min for occludin and fixed in ethanol for 30 min at room temperature and in acetone at −20°C for 5 min for ZO-1. Non-specific background was blocked by incubation with 10% bovine serum albumin (BSA) in Tris-buffered saline (TBS) and 0.3% Triton X-100 (30 min at room temperature). Sections were incubated with rabbit anti-occludin or rabbit anti-ZO-1 (1:400 for ZO-1 and 1:100 for occludin staining; Zymed Laboratories, San Francisco, California, USA) for 2 h. Sections were washed three times for 10 min in TBS and probed with goat anti-rabbit fluorescein isothiocyante (FITC)-conjugated antibodies (1:50, Zymax; Zymed Laboratories). Slides were washed three times for 10 min in TBS and mounted in mounting medium (Vectashield; Vector Laboratories, Burlingame, California, USA). Sections were visualised on a fluorescence microscope using a ×40 objective, and images were stored digitally with Leica software. As a control, slides were incubated with serial dilutions of the primary antibody to signal extinction. Two negative controls were used: slides incubated with irrelevant antibody or without primary antibody. All the stainings were performed in duplicate in non-serial distant sections, and analysed in a double-blind manner by two different investigators.

### Intestinal permeability in vivo

This measure is based on the intestinal permeability towards 4000 Da fluorescent dextran–FITC (DX-4000–FITC) (FD4000; Sigma-Aldrich, St. Louis, Missouri, USA) as described.^6^ ^43^ Briefly, mice that had fasted for 6 h were given DX-4000–FITC by gavage (500 mg/kg body weight, 125 mg/ml). After 1 h and 4 h, 120 μl of blood was collected from the tip of the tail vein. The blood was centrifuged at 4°C, 12 000 *g* for 3 min. Plasma was diluted in an equal volume of PBS (pH 7.4) and analysed for DX-4000–FITC concentration with a fluorescence spectrophotometer (HTS-7000 Plus-plate-reader; Perkin Elmer, Wellesley, Massachusetts, USA) at an excitation wavelength of 485 nm and emission wavelength of 535 nm. Standard curves were obtained by diluting FITC–dextran in non-treated plasma diluted with PBS (1:3 v/v).

### Biochemical analyses

Plasma LPS concentration was determined by using a kit based upon a *Limulus* amoebocyte extract (LAL kit endpoint-QCL1000; Cambrex BioScience, Walkersville, Maryland, USA), samples were diluted 1/40 to 1/100 and heated for 20 cycles of 10 min at 68°C and 10 min at 4°C. An internal control for LPS recovery was included in the calculation. Plasma cytokines (interleukin (IL) 1α, IL1b, tumour necrosis factor (TNF) α, IL6, monocyte chemoattractant protein (MCP)-1, macrophage inflammatory protein (MIP)-1α, IL10, interferon (INF) γ, IL15, IL18) and gut hormones (GLP-1 (active), GIP (total), amylin (active), pancreatic polypeptide) were respectively determined in duplicate by using a Bio-Plex Multiplex kit (Bio-Rad, Nazareth, Belgium), or a mouse gut hormones panel (LincoPlex; Millipore, Brussels, Belgium), and measured by using Luminex technology (Bio-Rad Bioplex; Bio-Rad) following the manufacturer’s instructions, an EIA kit (GLP-2 EIA kit) (Yanaihara Institute, Shizuoka, Japan) was used to quantify GLP-2.

### Statistical analysis

Results are presented as mean with the SEM. The statistical significance of differences was analysed by one-way ANOVA followed by post hoc Bonferroni’s multiple comparison test or Kruskal–Wallis for non-parametric data followed by Dunn’s multiple comparison test. Data with different superscript letters are significantly different p*<*0.05, according to the post hoc ANOVA statistical analysis. Comparisons between GLP-2-treated mice and control mice were performed using the two-tailed Student t test. Multiple correlation analyses were assessed by the Pearson’s test using GraphPad Prism version 5.00 for windows. Results were considered statistically significant when p*<*0.05.

## RESULTS

### Prebiotic treatment induces changes in the gut microbiota of *ob/ob* mice

Feeding *ob*/*ob* mice with the prebiotic carbohydrates (Ob-Pre) induced significant changes in the gut microbiota of the caecum, with a higher total bacteria count, *Lactobacillus* spp., *Bifidobacterium* spp., and the *C coccoides–E rectale* cluster (table 1), as compared to the control mice (Ob-CT and Ob-Cell).

**Figure 1:**
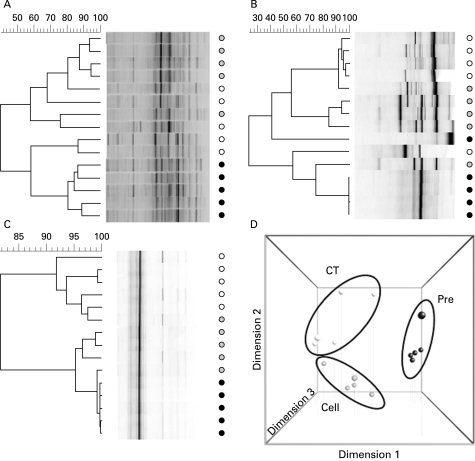
Prebiotic-associated changes in the gut microbiota. DGGE fingerprint patterns of the caecal microbial community of selected *ob*/*ob* mice fed a normal-diet (Ob-CT, white symbols), non-prebiotic control diet (Ob-Cell, grey symbols) or prebiotic diet (Ob-Pre, black symbols) for 5 weeks. The DGGE profiles were constructed using primers for (A) Total bacteria (B) *Bifidobacterium* spp (C) *Lactobacillus* spp. Cluster analysis is based on the Pearson product–moment correlation coefficient and UPGMA linkage. (D) Three-dimensional multidimensional scaling analysis conducted on the DGGE fingerprinting composite data set (total bacteria, *Bifidobacterium* spp. and *Lactobacillus* spp). CT, Cell and Pre respectively refer to the selected *ob*/*ob* mice fed a normal-diet, non-prebiotic control diet or prebiotic diet for 5 weeks. DGGE, denaturing gradient gel electrophoresis; UPGMA, unweighted pair group method with arithmetic mean clustering algorithm.

Changes in the microbial community composition were also observed upon DGGE analysis of the caecal content of the different treatment groups (fig 1A–C). Clustering of the DGGE fingerprints for total bacteria and for the specific groups of bifidobacteria and lactobacilli, indicated, for all fingerprints, a separate cluster of the Ob-Pre mice. Secondary clustering was observed between the Ob-CT and Ob-Cell mice. Finally, multidimensional scaling (MDS) analysis, performed on the composite data set derived from the combination of all the DGGE analyses of this study (total bacteria, lactobacilli and bifidobacteria), also indicated a specific clustering pattern, depending on the treatment of the *ob*/*ob* mice. (fig 1D). As the distance between 2 data points in the three-dimensional MDS plot is a visual representation of the difference in the microbial community composition of the caecum of the different mice, MDS analysis provides the final confirmation that the prebiotic treatment induced important changes in the gut microbiota of the Ob-Pre mice compared to the control groups.

### Changes in the gut microbiota upon prebiotic administration reduce intestinal permeability

**Figure 2:**
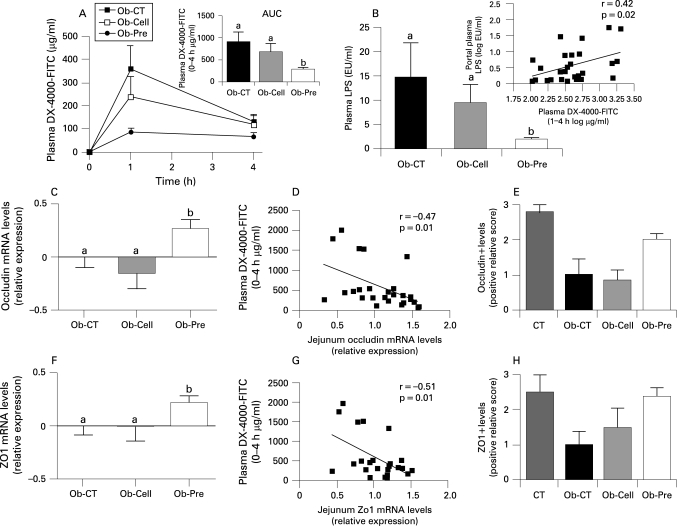
Prebiotic treatment reduces intestinal permeability. (A) Intestinal permeability assay: Plasma DX-4000–FITC (μg/ml) oral challenge measured in *ob/ob* mice fed a normal diet (Ob-CT), non-prebiotic control diet (Ob-Cell), prebiotic diet (Ob-Pre) for 5 weeks. The inset corresponds to the area under curve (AUC) in the same groups. (B) Plasma endotoxin (LPS) concentrations (EU/ml); the inset corresponds to correlation between plasma LPS levels and plasma DX-4000–FITC (Pearson’s r correlation and corresponding p value). (C,F) Jejunum epithelial tight-junction protein markers (ZO-1 and occludin mRNA concentrations) relative expression to Ob-CT. Data are mean with the SEM. Data with different superscript letters are significantly different (p*<*0.05), according to the post hoc ANOVA statistical analysis. (D,G) Correlations between intestinal permeability markers: plasma DX-4000–FITC and ZO-1 and occludin mRNA concentrations (p*<*0.05); the inset corresponds to Pearson’s r correlation and corresponding p value. (E,H) Immunohistochemistry score of the jejunum epithelial tight-junction proteins (ZO-1 and occludin) in wild-type (CT), Ob-CT, Ob-Cell or Ob-Pre mice. DX-4000, dextran of molecular weight 4000 Da; EU, endotoxin unit; FITC, fluroescein isothiocyanate; LPS, lipopolysaccharide.

**Figure 3:**
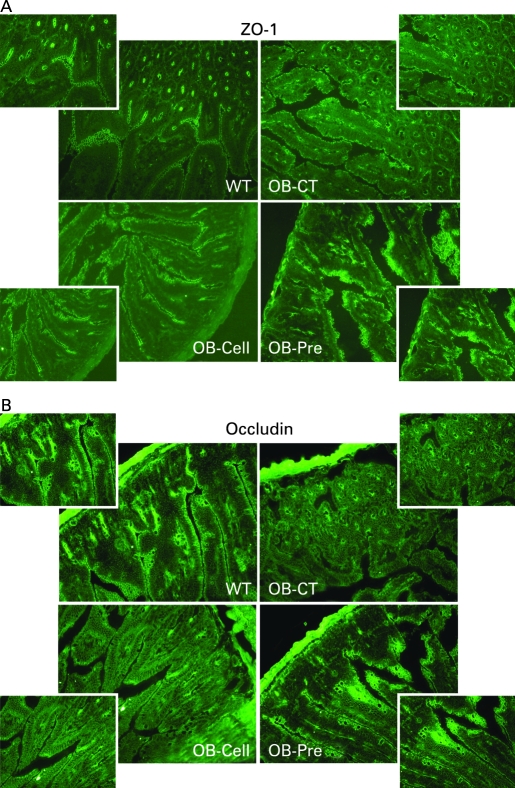
Prebiotic treatment changes tight-junction proteins distribution. Representative immunofluorescence staining for (A) ZO-1 and (B) occludin in *ob/ob* mice fed a normal diet (Ob-CT), non-prebiotic control diet (Ob-Cell), prebiotic diet (Ob-Pre) for 5 weeks. WT, wild-type.

Ob-Pre fed mice exhibited a 3-fold lower plasma DX-4000–FITC area under the curve (fig 2A) as compared to Ob-CT and Ob-Cell mice. In accordance with the in vivo assessment of intestinal permeability, LPS levels were significantly lower in Ob-Pre mice plasma samples, as compared to the other groups (fig 2B). Moreover, plasma DX-4000–FITC and portal plasma LPS levels were positively and significantly correlated (fig 2B, inset), further confirming the relation between intestinal permeability and the development of metabolic endotoxaemia. Gut permeability is controlled by several specific tight-junction proteins. Among these, ZO-1 and occludin have been proposed as key markers of tight-junction integrity.^26^ The prebiotic treatment increased ZO-1 and occludin mRNA in the jejunum segment (fig 2C,F). As previously suggested, a representative immunofluorescence assay performed on intestinal sections of wild-type C57BL/6J mice demonstrated an intact network of ZO-1 and occludin proteins which were predominantly localised along the apical cellular border.^26^ In strong contrast, ZO-1 and occludin staining appears to be decreased and discontinuous in Ob-CT tissues (fig 3A,B). Furthermore, immunohistochemical score analysis confirmed the strong alteration of ZO-1 and occludin distribution and expression as compared to wild-type mice (fig 2E,H). In accordance with the mRNA analysis, prebiotic feeding improved the tight junctions, since the immunohistochemical staining of both proteins localised along the apical cellular border was higher in Ob-Pre as compared to Ob-CT and Ob-Cell mice (fig 3A,B).

To identify whether the changes in the gut microbiota and tight-junction protein expression are associated with in vivo gut permeability, multiple correlation analysis between these parameters was performed. Indeed, a negative correlation between tight-junction proteins mRNA and plasma DX-4000–FITC was found in the jejunum segment (fig 2D,G), and in the colon (supplementary data 1). In addition, the caecal content of *Bifidobacterium* spp. and portal plasma levels of LPS showed a negative correlation (supplemental data 1), suggesting that the specific increase of bifidobacteria positively impacted the development of metabolic endotoxaemia.

### Changes in the gut microbiota upon prebiotic administration are associated with improved systemic and hepatic inflammation

**Figure 4:**
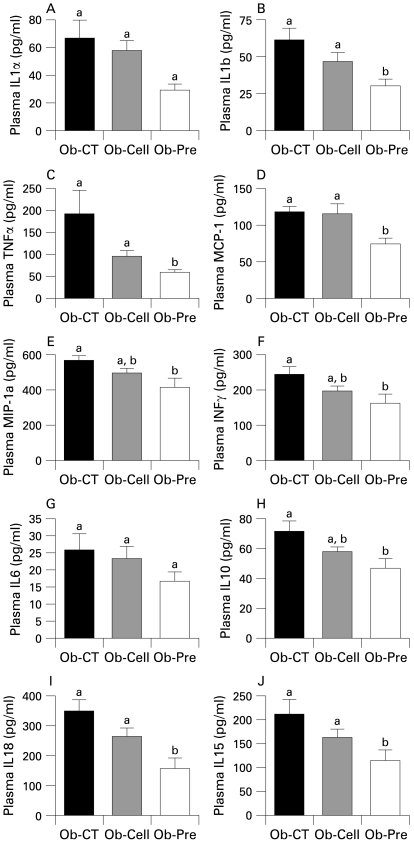
Prebiotic treatment reduces the occurrence of systemic inflammation. (A) IL1α, (B) IL1b, (C) TNFα, (D) MCP-1, (E) MIP-1a, (F) INFγ, (G) IL6, (H) IL10, (I) IL18 and (J) IL15 plasma levels (pg/ml) in *ob/ob* mice fed a normal diet (Ob-CT), non-prebiotic control diet (Ob-Cell) or prebiotic diet (Ob-Pre) for 5 weeks. Data are mean with the SEM. Data with different superscript letters are significantly different (p*<*0.05), according to the post hoc ANOVA statistical analysis. IFN, interferon; IL, interleukin; MCP-1, monocyte chemoattratant protein-1; MIP-1a, macrophage inflammatory protein-1a; TNF, tumour necrosis factor.

**Figure 5:**
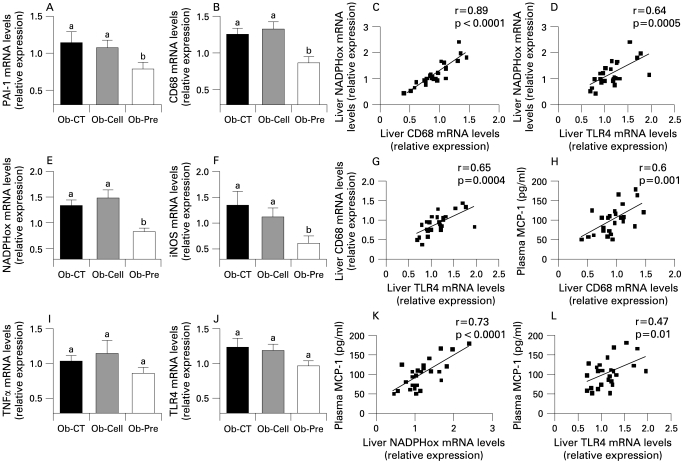
Changes in the gut microbiota control hepatic inflammation, oxidative stress and macrophage infiltration markers. (A,I) Inflammation: PAI-1, TNFα mRNA concentrations; (B,J) Macrophage infiltration markers: CD68, TLR4 mRNA concentrations; (E,F) Oxidative stress markers: NADPHox, iNOS mRNA concentrations in *ob/ob* mice fed a normal diet (Ob-CT), non-prebiotic control diet (Ob-Cell) or prebiotic diet (Ob-Pre) for 5 weeks. Data are mean with the SEM. Data with different superscript letters are significantly different (p*<*0.05), according to the post hoc ANOVA statistical analysis. Correlations between liver NADPHox mRNA and (C) liver CD68 mRNA, (D) TLR4 mRNA or (K) plasma MCP-1 levels. Correlations between plasma MCP-1 and (H) liver CD68 mRNA or (L) TLR4 mRNA. (G) Correlation between liver CD68 mRNA and TLR4 mRNA concentrations (p*<*0.05) the inset corresponds to Pearson’s r correlation and corresponding p value. iNOS, inducible nitric oxide synthase; MCP-1, monocyte chemoattractant protein-1; NADPHox, NADPH oxidase; PAI-1, plasminogen activator inhibitor; TLR, toll-like receptor; TNF, tumour necrosis factor.

To study the effects of changes in the gut microbiota and improved barrier function on the one hand, and metabolic disorders on the other hand, we first assessed systemic inflammation. All plasma cytokine and chemokine concentrations were decreased in Ob-Pre mice when compared to Ob-CT and Ob-Cell mice (fig 4). Metabolic endotoxaemia is frequently associated with hepatic inflammation, and with increased oxidative stress driving metabolic disorders. Here we found that changing the gut microbiota significantly reduced plasminogen activator inhibitor 1 (PAI-1), CD68, NADPH oxidase (NADPHox) and inducible nitric oxide synthase (iNOS) mRNA concentrations (fig 5A,B,E,F), and tended to decrease toll-like receptor 4 (TLR4) and TNFα mRNA concentrations (fig 5I,J). The oxidative stress marker NADPHox positively correlated with macrophage infiltration markers (CD68, TLR4) (fig 5 C,D). Moreover, both macrophage infiltration markers were correlated (fig 5G). Plasma chemokine MCP-1 positively correlated with tissue macrophages infiltration and oxidative stress markers (fig 5 H,K,L). Accordingly, plasma LPS levels were positively correlated with NADPHox and macrophage infiltration markers (liver CD68 mRNA and plasma MCP-1 levels) (supplemental data 1). Furthermore, we found that these markers were negatively correlated with the content of *Bifidobacterium* spp. (supplemental data 1). The lower inflammatory and oxidative stress was associated with a lower total hepatic lipid content (Ob-CT: 839^a^ (SEM 111); Ob-Cell: 781^a^ (SEM 42); Ob-Pre: 531^b^ (SEM 58) mg of lipids/liver, Ob-Pre p*<*0.05 vs Ob-CT and Ob-Cell) and confirmed by histological analysis (not shown). (Different superscript letters denote a statistical difference between the groups.) Altogether, these multiple correlations support a strong relationship between gut microbiota, gut permeability, systemic and hepatic inflammation, oxidative stress and macrophage infiltration in *ob/ob* mice.

### Prebiotic feeding modulates plasma gut peptides and adiposity

Prebiotic administration decreased food intake (Ob-CT: 24.9^a^ (SEM 0.7); Ob-Cell: 20.0^b^ (SEM 0.5); Ob-Pre: 16.3^c^ (SEM 1.1) kcal/day.mouse; p*<*0.05). We have previously demonstrated that prebiotic-induced changes in the gut microbiota modulate gastrointestinal peptides (GLP-1, peptide YY (PYY), ghrelin) involved in glucose homeostasis, appetite and/or body weight regulation.^8^ ^33^ ^44^^–^48 Here, we found that the prebiotic treatment also modulated portal plasma gut peptides in *ob/ob* mice, leading to a significant increase in GLP-1 (table 2). In addition, the glucose-dependent insulinotropic polypeptides (GIPs), another peptide associated with the development of obesity, diabetes and adiposity, was significantly decreased (table 2).^49^^–^51 In accordance with this, Ob-Pre mice had lower visceral, epididymal and subcutaneous adipose depots and a higher muscle mass as compared to the other groups (supplemental data 2).

**Table 2:** Changes in the gut microbiota upon administration of prebiotics impacts on portal plasma gut peptides

Gut peptide	Ob-CT	Ob-Cell	Ob-Pre
GLP-1 (active) (pmol/l)	7.65^a^ (1.52)	7.71^a^ (1.62)	21.15^b^ (6.71)
GIP (total) (pg/ml)	207.91^a^ (37.76)	180.71^a^ (27.53)	114.02^b^ (16.92)
Pancreatic polypeptide (pg/ml)	68.07^a^ (8.45)	75.94^a^ (6.56)	50.05^b^ (2.67)
Amylin (active) (pg/ml)	358.87^a^ (36.12)	364.99^a^ (45.07)	504.90^b^ (58.34)
GLP-2 (ng/ml)	0.56^a^ (0.06)	0.62^a^ (0.04)	0.75^b^ (0.05)

Data are mean (SEM).

Data with different superscript letters are significantly different at p*<*0.05, according to the post hoc ANOVA statistical analysis.

CT, Cell and Pre, respectively, refer to the selected *ob/ob* mice, fed a normal diet, non-prebiotic control diet or prebiotic diet. GIP, glucose-dependent insulinotropic polypeptide; GLP, glucogon-like peptide.

This study is the first in which it is shown that, besides an effect on gut peptides, feeding prebiotics also modulated two pancreatic peptides, since it significantly increased amylin (table 2) and decreased pancreatic polypeptide (PP) as compared to Ob-CT and Ob-Cell mice (table 2). The link between the above-mentioned peptides modulated by prebiotics and gut permeability has not been described so far. Moreover, the decrease in food intake and/or a decrease in adiposity are not involved in changes in gut permeability. Thus, we have hypothesised that GLP-2, a peptide clearly related to GLP-1 production, could link changes in gut microbiota and intestinal barrier function.

### Prebiotic administration increases intestinal proglucagon mRNA and portal plasma proglucagon-derived peptide GLP-2

**Figure 6:**
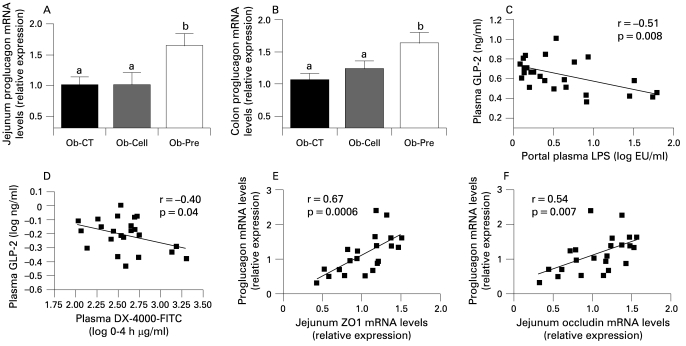
Prebiotic administration increases intestinal proglucagon mRNA and correlates with intestinal permeability markers. (A) Jejunum proglucagon mRNA concentrations; (B) proximal colon proglucagon mRNA concentrations in *ob/ob* mice fed a normal diet (Ob-CT), non-prebiotic control diet (Ob-Cell), prebiotic diet (Ob-Pre) for 5 weeks. Data are mean with the SEM. Data with different superscript letters are significantly different (p*<*0.05), according to the post hoc ANOVA statistical analysis. Correlations between plasma GLP-2 levels and (C) plasma LPS plasma; or (D) DX-4000–FITC plasma levels. Correlations between proglucagon mRNA and (E) jejunum ZO-1 or (F) occludin mRNA concentrations; the inset corresponds to Pearson’s r correlation and corresponding p value. DX 4000, dextran of molecular weight 4000 Da; FITC, fluorescein isothiocyanate; GLP-2, glucogon-like protein-2; LPS, lipopolysaccharide.

We and other have previously shown that changing gut microbiota by using fermentable non-digestible carbohydrate significantly increases gut weight and promotes proliferation of epithelial cells.^52^^–^54 We have also shown – and confirmed in the present study (table 2) – that feeding mice with prebiotic doubled GPL-1 portal plasma levels.^8^ ^33^ ^44^ ^45^ ^47^ ^48^ ^55^ ^56^ In previous studies, the increase in GLP-1 plasma level was associated with a higher GPL-1 peptide and proglucagon mRNA expression in the (proximal) colon tissue. Interestingly, our study is the first to show that a higher proglucagon mRNA content occurs in the proximal colon – as expected – and also in the jejunum (fig 6A,B). Among the proglucagon-derived peptides, GLP-2 is co-secreted with GLP-1 and exerts an intestinotrophic effect.^39^ ^57^ ^58^ GLP-2 enhances intestinal epithelial proliferation and reduces gut permeability,^59^^–^62 hence, we found that Ob-Pre fed mice exhibited a significant increase of plasma GLP-2 levels (table 2). Importantly, portal plasma GLP-2 and both plasma markers of gut permeability (DX-4000–FITC and LPS) were negatively correlated (fig 6C,D). Along the same line, intestinal proglucagon and tight-junction mRNA (ZO-1, occludin) were positively correlated (fig 6E,F). Altogether, these data suggest that the prebiotic-induced intestinal proglucagon mRNA enhanced expression and the consequent GLP-2 production indicate that this peptide may positively impacts on gut barrier integrity.

### Chronic GLP-2 antagonist treatment blunts prebiotic-induced reduction in endotoxaemia and hepatic inflammatory tone

To causally link changes in endogenous GLP-2 production upon prebiotic treatment with the improvement of metabolic endotoxaemia, and inflammatory markers, we chronically blocked GLP-2 receptor in prebiotic fed *ob/ob* mice by using GLP-2 antagonist.

**Figure 7:**
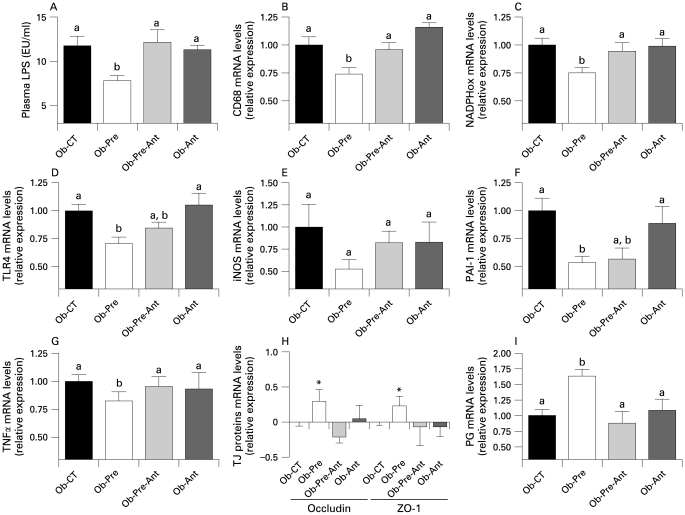
Chronic GLP-2 antagonist treatment blunts prebiotic-induced reduction in endotoxaemia and hepatic inflammatory tone. (A) Plasma LPS concentrations (EU/ml); (B,D) Macrophage infiltration markers: TLR4, CD68mRNA concentrations; (C,E) oxidative stress markers: NADPHox, iNOS mRNA concentrations; (F,G) hepatic inflammation: PAI-1, TNFα mRNA concentrations; (H) jejunum epithelial tight-junction protein markers (ZO-1 and occludin mRNA) concentrations; (I) jejunum proglucagon mRNA concentrations in *ob/ob* mice fed a normal diet and injected twice daily with a saline (Ob-CT), prebiotic diet and injected twice daily with a saline (Ob-Pre), prebiotic diet and injected twice daily with GLP-2 antagonist (ObPre-Ant), normal diet and injected twice daily with GLP-2 antagonist (Ob-Ant) for 4 weeks. Data are mean with the SEM. *Significantly different (p*<*0.05) from Ob-CT and Ob-Pre-Ant according to the Kruskal–Wallis test analysis. Data with different superscript letters are significantly different (p*<*0.05), according to the post hoc ANOVA statistical analysis. EU, endotoxin unit; GLP-2, glucagon-like protein-2; iNOS, inducible nitric oxide synthase; NADPHox, NADPH oxidase; PAI-1, plasminogen activator inhibitor-1; PG, proglucagon; TJ, tight junction; TLR, toll-like receptor; TNF, tumour necrosis factor.

In this second set of experiments, we confirm the data observed in the first study showing that prebiotic-induced changes in gut microbiota are linked to a significant decrease in plasma LPS levels, hepatic inflammation and oxidative stress markers such as PAI-1, CD68, NADPHox and TLR4 mRNA concentrations (fig 7B–D,F), and to a tendency to decrease for iNOS and TNFα mRNA concentrations (fig 7E,G). In addition, we also confirm that the prebiotic treatment increases ZO-1 and occludin mRNA (fig 7H) and the GLP-2 precursor transcript (proglucagon mRNA) content in the jejunum (fig 7I).

Importantly, the GLP-2 antagonist completely blunts the prebiotic-induced reduction endotoxaemia (fig 7A). Hence, most of the prebiotic-induced changes of hepatic inflammation and oxidative stress markers are also controlled by a GLP-2-dependent mechanism. We observed that mice treated concomitantly with the prebiotic and the GLP-2 antagonist or GLP-2 antagonist alone exhibit CD68, NADPHox and TLR4 mRNA levels (fig 7B–D) similar to the control mice, except for the PAI-1 mRNA levels (fig 7F) which remains equivalent to the prebiotic treated mice with or without GLP-2 antagonist treatment. Moreover, GLP-2 antagonist treatment completely blocked the enhanced proglucagon, ZO1 and occludin mRNA levels found in prebiotic fed mice that reach values similar to the control (fig 7H,I).

### Chronic GLP-2 pharmacological treatment lowers endotoxaemia, improves gut permeability markers, and reduces systemic and hepatic inflammation, oxidative stress and macrophage infiltration markers

To study the impact of a pharmacological GLP-2 treatment, without modulation of the gut microbiota, we treated *ob/ob* mice with GLP-2 for 12 days. Body weight, food intake, adiposity, liver weight were equivalent between Ob-CT mice and Ob-GLP-2 mice (supplemental data 3).

**Figure 8:**
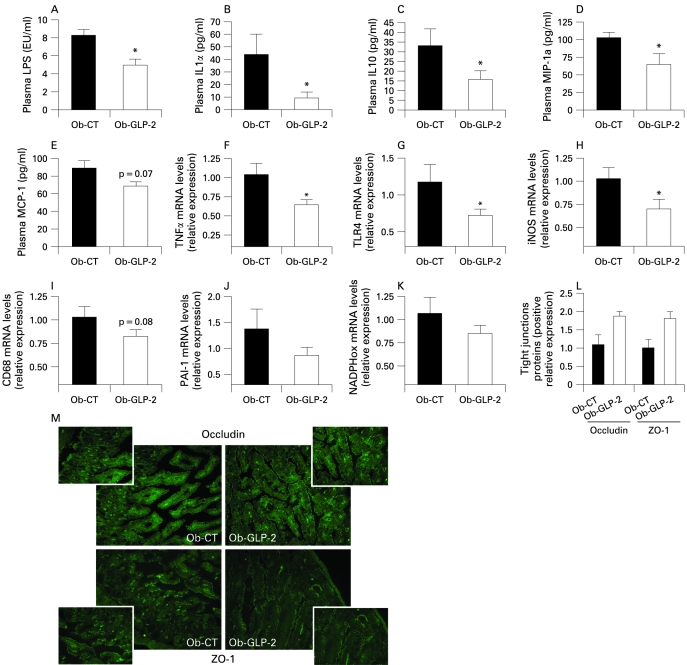
Chronic GLP-2 treatment lowers endotoxaemia, improves gut permeability markers, and reduces systemic and hepatic inflammation, oxidative stress and macrophage infiltration markers. (A) Plasma LPS concentrations (EU/ml); Cytokines and chemokines plasma levels (pg/ml): (B) IL1α; (C) IL10; (D) MIP-1a; (E) MCP-1; (F,J) hepatic inflammation: TNFα, PAI-1 mRNA concentrations; (G,I) macrophage infiltration markers: TLR4, CD68mRNA concentrations; (H,K) oxidative stress markers: iNOS, NADPHox mRNA; (L) immunohistochemistry score and (M) representative immunofluorescence staining of the jejunum epithelial tight-junction proteins (ZO-1 and occludin) measured in *ob/ob* mice injected twice daily with a saline vehicle (Ob-CT) or GLP-2 (Ob-GLP-2) for 12 days. Data are mean with the SEM. *Significantly different (p*<*0.05) from Ob-CT mice according to the two-tailed Student t test. EU, endotoxin unit; GLP-2, glucogon-like protein-2; iNOS, inducible nitric oxide synthase; IL, interleukin; LPS, lipopolysaccharide; MCP-1, monocyte chemoattractant protein-1; MIP-1a, macrophage inflammatory protein-1a; NADPHox, NADPH oxidase; TLR, toll-like receptor; TNF, tumour necrosis factor.

Importantly, we demonstrated that GLP-2 administration to *ob/ob* mice dramatically decreased plasma LPS by about 50% (fig 8A). This was accompanied by a lower plasma inflammatory tone (IL1a, MIP-1a, MCP-1, IL10) (fig 8B–E)). Furthermore, as reported in both prebiotics-fed mice studies, mRNA concentrations of macrophage infiltration (TLR4, CD68) (fig 8G,I), inflammation (TNFα, PAI-1) (fig 8F,J) and oxidative stress (iNOS, NADPHox) markers (fig 8H,K) were decreased in Ob-GLP-2 mice as compared to Ob-CT mice. Finally, ZO-1 and occludin staining immunohistochemical score analysis confirmed the strong impact of GLP-2 on the tight-junctions proteins (fig 8L). In accordance with the prebiotic effect, the immunohistochemical staining of both proteins localised along the apical cellular border was higher in Ob-GLP-2 as compared to Ob-CT (fig 8M).

Therefore, the gut peptide GLP-2 appears as a credible link between gut microbiota, gut permeability, and inflammatory tone.

## DISCUSSION

Several reports have demonstrated that the gut microbiota participates in the development of obesity by several mechanisms, ie, by harvesting energy from the diet, by modulating the synthesis of gut peptides involved in energy homeostasis, and/or by regulating fat storage.^63^ We have recently demonstrated that mice fed a high-fat diet were characterised by an increase in gut permeability and metabolic endotoxaemia. The inflammatory phenotype of *ob/ob* mice originates from the gut microbiota, since changing the gut microbiota using antibiotics lowers endotoxaemia-induced inflammation and metabolic disorders.^6^ ^64^ ^65^ Recently, it has been proposed that this higher endotoxaemia in *ob/ob* mice and *db/db* mice was dependent on a disruption of the key tight-junction proteins, ZO-1 and occludin.^26^ Here we confirm that obese mice exhibit an altered gut barrier, characterised by a disruption of tight-junction proteins. Among the mechanisms involved in this phenomenon, excessive TNFα production was proposed to help propagate the extension of a local or systemic inflammation and this inflammatory process per se would trigger the alteration of both junctional proteins.^24^ Our data show that prebiotic-fed mice have a lower level of several plasma cytokines well-known to promote tight-junction disruption such as TNFα, IL1b, IL1α, IL6, INFγ.^66^^–^69 In addition, we also found a significant decrease of the chemokines MCP-1 and MIP-1a.

In the present study, we demonstrated, in two separate set of experiments, that changing gut microbiota of *ob/ob* mice in favour of the *Bifidobacterium* spp. is associated with a significant improvement of gut permeability measured in vivo; this phenomenon was linked to an increase in tight-junction mRNA expression and proteins distribution. The improved gut barrier observed in prebiotic-fed mice was correlated to the lower portal plasma LPS levels and inflammatory tone (ie, decrease in circulating cytokines). The leakage of gut microbiota-derived LPS into the portal blood is a well-established mechanism of metabolic endotoxaemia that triggers liver inflammation and oxidative stress.^26^ ^70^ In the present studies, we found that lowering systemic inflammation by prebiotics is significantly correlated with a strong decrease in markers of oxidative and inflammatory stress in the liver tissue. Altogether, these data strongly suggest that the modulation of the gut microbiota using prebiotics in obese mice, could act favourably on the intestinal barrier, thereby reducing endotoxaemia, systemic and liver inflammation, with beneficial consequences on associated metabolic disorders.

However, the mechanisms by which prebiotics improve gut permeability – in the particular context of obesity – are not fully understood. One potential explanation could arise from the putative role of the *Bifidobacterium* spp in maintaining the gut barrier. Interestingly, *Bifidobacterium* spp do not degrade intestinal mucus glycoproteins like other pathogenic bacteria do, and *Bifidobacterium* spp promote a healthier microvillus environment by preventing permeability and bacterial translocation.^71^ ^72^ Among the putative mechanisms, it has been demonstrated that the prebiotic modulation of the gut microbiota increases villus height and crypt depth, and leads to a thicker mucosal layer in the jejunum and in the colon.^73^ These effects are related to the bacterial fermentation of the prebiotics. The resulting products of fermentation are mainly short-chain fatty acids (SCFAs), with butyrate acting as an energetic substrate for the colonocytes and having a trophic effect on mucosa.^74^ ^75^ In epithelial monolayers model, it has been shown that other probiotics strains such as *Lactobacillus rhamnosus* GG and *Lactobacillus* *casei* DN-114-001 protect epithelial barrier function against *Escherichia coli*-induced redistribution of the tight-junction proteins.^76^ ^77^ Besides the putative role of the SCFAs and specific bacterial strains, the exact mechanism underlying the relation between prebiotic-induced changes in gut microbiota and improved gut barrier function has not been described so far.

Here we found that changes in the gut microbiota of *ob/ob* mice exert a trophic effect on the intestine of Ob-Pre mice, a phenomenon associated with a higher proglucagon mRNA expression in both the jejunum and colon. We have previously demonstrated that prebiotic-induced changes in the gut microbiota promote GLP-1 synthesis (proglucagon mRNA and GLP-1 peptide content) in the proximal colon by a mechanism linked to the differentiation of precursor cells into enteroendocrine cells.^33^ ^44^^–^48 In the present study, we additionally found that changing gut microbiota using prebiotics is associated with an increased endogenous production of the glucagon-like peptide-2 (GLP-2). GLP-2 is a 33 amino acid peptide, formed, as GLP-1, from the cleavage of proglucagon peptide.^57^ GLP-2 is associated with intestinal growth and adaptation in a variety of pathological conditions, including post-resection intestinal adaptation, coeliac disease, parental nutrition-induced intestinal atrophy and inflammatory bowel disease.^78^^–^80 In addition, GLP-2 has also been demonstrated to enhance intestinal adaptation^57^ and to reduce the internalisation of enteric bacteria in INT-407 enterocytes in vitro model.^81^ Strikingly, the intestinotrophic effects observed following prebiotic administration are similar to the physiological effect of GLP-2. The increased GLP-2 production is in accordance with our earlier study, where we found that changing the gut microbiota of rats fed a high-fat diet following a prebiotic treatment, doubled intestinal GLP-2 concentrations.^46^ Besides, here we found that the higher endogenous GLP-2 production is associated with an improvement of the mucosal barrier function, leading to improved tight junctions and decreased plasma LPS concentrations and therefore blunted inflammatory and oxidative stresses. A recent report demonstrates that a chronic treatment with GLP-2 protects rats against the ischaemia–reperfusion-induced endotoxaemia and inflammation, by an unknown mechanism.^82^

Our first experiment strongly suggests that the lower metabolic endotoxaemia and the consequent low-grade inflammation observed upon prebiotic feeding could be a mechanism involving an enhanced endogenous GLP-2 production. Indeed, we found that Ob-Pre fed mice exhibited a significant increase of plasma GLP-2 levels and that this parameter negatively correlates with markers of gut permeability. Along the same line, we found a positive correlation between intestinal proglucagon and tight-junction proteins mRNA (ZO-1, occludin). Altogether, these data led us to postulate that the prebiotic-induced intestinal proglucagon mRNA expression and the consequent GLP-2 production may positively impact on gut barrier integrity. Therefore, to investigate this hypothesis, we performed a second set of experiments where we treated mice concomitantly with the prebiotic and a GLP-2 antagonist. First, we confirm the improvement of inflammatory markers following the prebiotic treatment; second, and more importantly, we show that the GLP-2 antagonist completely blocks the major features of the prebiotic treatment. Hence, in absence of GLP-2 signalling, the prebiotic treatment failed to reduce plasma LPS levels, and most of the hepatic inflammatory and oxidative stress markers remained equivalent to the control mice. In addition, the GLP-2 antagonist blocks the positive effects of the prebiotic treatment on both intestinal tight-junction proteins and proglucagon mRNA. Altogether, both set of experiments strongly support that specific changes in gut microbiota improve gut permeability and inflammatory tone by a GLP-2 dependent mechanism.

To support the putative therapeutic effect of GLP-2 in the context of a low-grade inflammation associated with obesity, and in order to delineate the protective role of the GLP-2 independently of the modulation of the gut microbiota, we chronically treated mice with GLP-2. This third set of experiment clearly shows that GLP-2 treatment alone significantly lowers inflammatory tone in obese mice. We found that GLP-2 treated mice exhibited a lower metabolic endotoxaemia, and a lower systemic and hepatic inflammatory tone. These features were associated with a strong positive effect of GLP-2 on the tight-junction proteins distribution. This set of experiments demonstrates that GLP-2 can be useful to treat the altered gut barrier observed in obesity and diabetes, and therefore could lower the inflammatory tone associated with these diseases. It would be interesting to see whether the magnitude of those effects is similar in other models such as diet-induced obesity and diabetes (ie, non-leptin-deficient models).

While the molecular and cellular mechanisms underlying the effect of GLP-2 on the tight junctions and the gut barrier function remained to define, several hypotheses can be postulated. GLP-2 increases the rate of crypt cell proliferation, villus elongation and reduces apoptosis, leading to improved barrier function.^83^ It has been proposed that insulin-like growth factor (IGF)-I acts as an essential target of GLP-2. Recent data have shown that mice lacking IGF-I were resistant to GLP-2-induced intestinal growth, crypt–villus height, and crypt cell proliferation. Furthermore, GLP-2 administration increases intestinal IGF-I secretion in vitro and enhances intestinal IGF-I mRNA transcript levels both in vitro and in vivo.^84^ In addition to this potential mechanism, data suggest that the downstream molecular mechanism by which GLP-2 receptor activation controls barrier function could be related to the activation of the β-catenin signalling pathway.^39^ Nonetheless, further studies are required to fully characterise the mechanisms through which GLP-2 improves gut barrier function.

Together, using the complementary approaches of the specific modulation of the gut microbiota, and of the pharmacological inhibition or activation of the GLP-2 receptor, our findings strongly suggest that GLP-2 participates to the modulation of the gut barrier function and the consequent systemic and hepatic inflammatory phenotype associated with obesity.

In addition, these data demonstrate that a selective modulation of the gut microbiota improves intestinal permeability and inflammatory markers during obesity and diabetes, via an unexpected mechanism such as higher GLP-2 endogenous production.

Thus, we propose that a selective gut microbiota modulation controls and increases endogenous production of the intestinotrophic proglucagon derived peptides GLP-2, and consequently improves gut barrier functions by a GLP-2-dependent mechanism. These new findings demonstrate the usefulness of developing specific therapeutic strategies using GLP-2 to tackle metabolic endotoxaemia and disorders associated with obesity and diabetes, and that gut microbiota modulation could be an interesting tool in this context.

## References

[1] Alberti KG Zimmet P Shaw J The metabolic syndrome – a new worldwide definition. Lancet 2005;366:1059–62 16182882 10.1016/S0140-6736(05)67402-8

[2] Matarese G Mantzoros C La CA Leptin and adipocytokines: bridging the gap between immunity and atherosclerosis. Curr Pharm Des 2007;13:3676–80 18220805 10.2174/138161207783018635

[3] Backhed F Manchester JK Semenkovich CF Mechanisms underlying the resistance to diet-induced obesity in germ-free mice. Proc Natl Acad Sci U S A 2007;104:979–84 17210919 10.1073/pnas.0605374104 PMC1764762

[4] Ley RE Backhed F Turnbaugh P Obesity alters gut microbial ecology. Proc Natl Acad Sci U S A 2005;102:11070–5 16033867 10.1073/pnas.0504978102 PMC1176910

[5] Backhed F Ding H Wang T The gut microbiota as an environmental factor that regulates fat storage. Proc Natl Acad Sci U S A 2004;101:15718–23 15505215 10.1073/pnas.0407076101 PMC524219

[6] Cani PD Bibiloni R Knauf C Changes in gut microbiota control metabolic endotoxemia-induced inflammation in high-fat diet-induced obesity and diabetes in mice. Diabetes 2008;57:1470–81 18305141 10.2337/db07-1403

[7] Cani PD Amar J Iglesias MA Metabolic endotoxemia initiates obesity and insulin resistance. Diabetes 2007;56:1761–72 17456850 10.2337/db06-1491

[8] Cani PD Neyrinck AM Fava F Selective increases of bifidobacteria in gut microflora improve high-fat-diet-induced diabetes in mice through a mechanism associated with endotoxaemia. Diabetologia 2007;50:2374–83 17823788 10.1007/s00125-007-0791-0

[9] Cai D Yuan M Frantz DF Local and systemic insulin resistance resulting from hepatic activation of IKK-beta and NF-kappaB. Nat Med 2005;11:183–90 15685173 10.1038/nm1166 PMC1440292

[10] Shi H Kokoeva MV Inouye K TLR4 links innate immunity and fatty acid-induced insulin resistance. J Clin Invest 2006;116:3015–25 17053832 10.1172/JCI28898 PMC1616196

[11] Poggi M Bastelica D Gual P C3H/HeJ mice carrying a toll-like receptor 4 mutation are protected against the development of insulin resistance in white adipose tissue in response to a high-fat diet. Diabetologia 2007;50:1267–76 17426960 10.1007/s00125-007-0654-8

[12] Song MJ Kim KH Yoon JM Activation of Toll-like receptor 4 is associated with insulin resistance in adipocytes. Biochem Biophys Res Commun 2006;346:739–45 16781673 10.1016/j.bbrc.2006.05.170

[13] Suganami T Mieda T Itoh M Attenuation of obesity-induced adipose tissue inflammation in C3H/HeJ mice carrying a Toll-like receptor 4 mutation. Biochem Biophys Res Commun 2007;354:45–9 17210129 10.1016/j.bbrc.2006.12.190

[14] Tsukumo DM Carvalho-Filho MA Carvalheira JB Loss-of-function mutation in Toll-like receptor 4 prevents diet-induced obesity and insulin resistance. Diabetes 2007;56:1986–98 17519423 10.2337/db06-1595

[15] Adachi Y Moore LE Bradford BU Antibiotics prevent liver injury in rats following long-term exposure to ethanol. Gastroenterology 1995;108:218–24 7806045 10.1016/0016-5085(95)90027-6

[16] Lichtman SN Keku J Schwab JH Hepatic injury associated with small bowel bacterial overgrowth in rats is prevented by metronidazole and tetracycline. Gastroenterology 1991;100:513–9 1985047 10.1016/0016-5085(91)90224-9

[17] Lichtman SN Sartor RB Keku J Hepatic inflammation in rats with experimental small intestinal bacterial overgrowth. Gastroenterology 1990;98:414–23 2295397 10.1016/0016-5085(90)90833-m

[18] Nanji AA Khettry U Sadrzadeh SM Severity of liver injury in experimental alcoholic liver disease. Correlation with plasma endotoxin, prostaglandin E2, leukotriene B4, and thromboxane B2. Am J Pathol 1993;142:367–73 8382006 PMC1886727

[19] Nishida J Ekataksin W McDonnell D Ethanol exacerbates hepatic microvascular dysfunction, endotoxemia, and lethality in septic mice. Shock 1994;1:413–8 7735970 10.1097/00024382-199406000-00004

[20] Enomoto N Ikejima K Yamashina S Kupffer cell sensitization by alcohol involves increased permeability to gut-derived endotoxin. Alcohol Clin Exp Res 2001;25 6 Suppl:51S–4S 11410742 10.1097/00000374-200106001-00012

[21] Enomoto N Ikejima K Bradford B Alcohol causes both tolerance and sensitization of rat Kupffer cells via mechanisms dependent on endotoxin. Gastroenterology 1998;115:443–51 9679050 10.1016/s0016-5085(98)70211-2

[22] Rivera CA Bradford BU Seabra V Role of endotoxin in the hypermetabolic state after acute ethanol exposure. Am J Physiol 1998;275 6 Pt 1:G1252–8 9843760 10.1152/ajpgi.1998.275.6.G1252

[23] Rivera CA Tcharmtchi MH Mendoza L Endotoxemia and hepatic injury in a rodent model of hindlimb unloading. J Appl Physiol 2003;95:1656–63 12794033 10.1152/japplphysiol.00302.2003

[24] Mazzon E Cuzzocrea S Role of TNF-alpha in ileum tight junction alteration in mouse model of restraint stress. Am J Physiol Gastrointest Liver Physiol 2008;294:G1268–80 18308862 10.1152/ajpgi.00014.2008

[25] Paulos CM Wrzesinski C Kaiser A Microbial translocation augments the function of adoptively transferred self/tumor-specific CD8 T cells via TLR4 signaling. J Clin Invest 2007;117:2197–204 17657310 10.1172/JCI32205 PMC1924500

[26] Brun P Castagliuolo I Leo VD Increased intestinal permeability in obese mice: new evidence in the pathogenesis of nonalcoholic steatohepatitis. Am J Physiol Gastrointest Liver Physiol 2007;292:G518–25 17023554 10.1152/ajpgi.00024.2006

[27] Turnbaugh PJ Backhed F Fulton L Diet-induced obesity is linked to marked but reversible alterations in the mouse distal gut microbiome. Cell Host Microbe 2008;3:213–23 18407065 10.1016/j.chom.2008.02.015 PMC3687783

[28] Wang Z Xiao G Yao Y The role of bifidobacteria in gut barrier function after thermal injury in rats. J Trauma 2006;61:650–7 16967002 10.1097/01.ta.0000196574.70614.27

[29] Griffiths EA Duffy LC Schanbacher FL In vivo effects of bifidobacteria and lactoferrin on gut endotoxin concentration and mucosal immunity in Balb/c mice. Dig Dis Sci 2004;49:579–89 15185861 10.1023/b:ddas.0000026302.92898.ae

[30] Wang ZT Yao YM Xiao GX Risk factors of development of gut-derived bacterial translocation in thermally injured rats. World J Gastroenterol 2004;10:1619–24 15162536 10.3748/wjg.v10.i11.1619 PMC4572765

[31] Commane DM Shortt CT Silvi S Effects of fermentation products of pro- and prebiotics on trans-epithelial electrical resistance in an in vitro model of the colon. Nutr Cancer 2005;51:102–9 15749636 10.1207/s15327914nc5101_14

[32] Ruan X Shi H Xia G Encapsulated Bifidobacteria reduced bacterial translocation in rats following hemorrhagic shock and resuscitation. Nutrition 2007;23:754–61 17706400 10.1016/j.nut.2007.07.002

[33] Cani PD Knauf C Iglesias MA Improvement of glucose tolerance and hepatic insulin sensitivity by oligofructose requires a functional glucagon-like peptide 1 receptor. Diabetes 2006;55:1484–90 16644709 10.2337/db05-1360

[34] Shin ED Estall JL Izzo A Mucosal adaptation to enteral nutrients is dependent on the physiologic actions of glucagon-like peptide-2 in mice. Gastroenterology 2005;128:1340–53 15887116 10.1053/j.gastro.2005.02.033

[35] Thulesen J Knudsen LB Hartmann B The truncated metabolite GLP-2 (3-33) interacts with the GLP-2 receptor as a partial agonist. Regul Pept 2002;103:9–15 11738243 10.1016/s0167-0115(01)00316-0

[36] Nelson DW Murali SG Liu X Insulin-like growth factor I and glucagon-like peptide-2 responses to fasting followed by controlled or ad libitum refeeding in rats. Am J Physiol Regul Integr Comp Physiol 2008;294:R1175–84 18256135 10.1152/ajpregu.00238.2007

[37] Thulesen J Hartmann B Hare KJ Glucagon-like peptide 2 (GLP-2) accelerates the growth of colonic neoplasms in mice. Gut 2004;53:1145–50 15247183 10.1136/gut.2003.035212 PMC1774162

[38] Kitchen PA FitzGerald AJ Goodlad RA Glucagon-like peptide-2 increases sucrase-isomaltase but not caudal-related homeobox protein-2 gene expression. Am J Physiol Gastrointest Liver Physiol 2000;278:G425–8 10712262 10.1152/ajpgi.2000.278.3.G425

[39] Dube PE Rowland KJ Brubaker PL Glucagon-like peptide-2 activates beta-catenin signaling in the mouse intestinal crypt: role of insulin-like growth factor-I. Endocrinology 2008;149:291–301 17884945 10.1210/en.2007-0561

[40] Van de Wiele T Boon N Possemiers S Prebiotic effects of chicory inulin in the simulator of the human intestinal microbial ecosystem. FEMS Microbiol Ecol 2004;51:143–53 16329863 10.1016/j.femsec.2004.07.014

[41] Boon N De Windt W Verstraete W Evaluation of nested PCR–DGGE (denaturing gradient gel electrophoresis) with group-specific 16S rRNA primers for the analysis of bacterial communities from different wastewater treatment plants. Fems Microbiol Ecol 2002;39:101–12 10.1111/j.1574-6941.2002.tb00911.x 19709189

[42] Possemiers S Bolca S Grootaert C The prenylflavonoid isoxanthohumol from hops (*Humulus lupulus* L.) is activated into the potent phytoestrogen 8-prenylnaringenin in vitro and in the human intestine. J Nutr 2006;136:1862–7 16772450 10.1093/jn/136.7.1862

[43] Wang Q Fang CH Hasselgren PO Intestinal permeability is reduced and IL-10 levels are increased in septic IL-6 knockout mice. Am J Physiol Regul Integr Comp Physiol 2001;281:R1013–23 11507020 10.1152/ajpregu.2001.281.3.R1013

[44] Cani PD Hoste S Guiot Y Dietary non-digestible carbohydrates promote L-cell differentiation in the proximal colon of rats. Br J Nutr 2007;98:32–7 17367575 10.1017/S0007114507691648

[45] Delzenne NM Cani PD Neyrinck AM Modulation of glucagon-like peptide 1 and energy metabolism by inulin and oligofructose: experimental data. J Nutr 2007;137:2547S–51S 17951500 10.1093/jn/137.11.2547S

[46] Cani PD Neyrinck AM Maton N Oligofructose promotes satiety in rats fed a high-fat diet: involvement of glucagon-like peptide-1. Obes Res 2005;13:1000–7 15976142 10.1038/oby.2005.117

[47] Cani PD Daubioul CA Reusens B Involvement of endogenous glucagon-like peptide-1(7-36) amide on glycaemia-lowering effect of oligofructose in streptozotocin-treated rats. J Endocrinol 2005;185:457–65 15930172 10.1677/joe.1.06100

[48] Cani PD Dewever C Delzenne NM Inulin-type fructans modulate gastrointestinal peptides involved in appetite regulation (glucagon-like peptide-1 and ghrelin) in rats. Br J Nutr 2004;92:521–6 15469657 10.1079/bjn20041225

[49] McClean PL Irwin N Cassidy RS GIP receptor antagonism reverses obesity, insulin resistance, and associated metabolic disturbances induced in mice by prolonged consumption of high-fat diet. Am J Physiol Endocrinol Metab 2007;293:E1746–55 17848629 10.1152/ajpendo.00460.2007

[50] Gault VA McClean PL Cassidy RS Chemical gastric inhibitory polypeptide receptor antagonism protects against obesity, insulin resistance, glucose intolerance and associated disturbances in mice fed high-fat and cafeteria diets. Diabetologia 2007;50:1752–62 17558485 10.1007/s00125-007-0710-4

[51] Althage MC Ford EL Wang S Targeted ablation of GIP-producing cells in transgenic mice reduces obesity and insulin resistance induced by a high fat diet. J Biol Chem 2008;283:18365–76 18420580 10.1074/jbc.M710466200 PMC2440595

[52] Reimer RA McBurney MI Dietary fiber modulates intestinal proglucagon messenger ribonucleic acid and postprandial secretion of glucagon-like peptide-1 and insulin in rats. Endocrinology 1996;137:3948–56 8756571 10.1210/endo.137.9.8756571

[53] Delzenne NM Kok N Deloyer P Dietary fructans modulate polyamine concentration in the cecum of rats. J Nutr 2000;130:2456–60 11015472 10.1093/jn/130.10.2456

[54] Kok NN Morgan LM Williams CM Insulin, glucagon-like peptide 1, glucose-dependent insulinotropic polypeptide and insulin-like growth factor I as putative mediators of the hypolipidemic effect of oligofructose in rats. J Nutr 1998;128:1099–103 9649591 10.1093/jn/128.7.1099

[55] Urias-Silvas JE Cani PD Delmee E Physiological effects of dietary fructans extracted from *Agave tequilana* Gto. and *Dasylirion* spp. Br J Nutr 2008;99:254–61 17711612 10.1017/S0007114507795338

[56] Delmee E Cani PD Gual G Relation between colonic proglucagon expression and metabolic response to oligofructose in high fat diet-fed mice. Life Sci 2006;79:1007–13 16757002 10.1016/j.lfs.2006.05.013

[57] Dube PE Brubaker PL Frontiers in glucagon-like peptide-2: multiple actions, multiple mediators. Am J Physiol Endocrinol Metab 2007;293:E460–5 17652153 10.1152/ajpendo.00149.2007

[58] Drucker DJ Erlich P Asa SL Induction of intestinal epithelial proliferation by glucagon-like peptide 2. Proc Natl Acad Sci U S A 1996;93:7911–6 8755576 10.1073/pnas.93.15.7911 PMC38848

[59] Jasleen J Ashley SW Shimoda N Glucagon-like peptide 2 stimulates intestinal epithelial proliferation in vitro. Dig Dis Sci 2002;47:1135–40 12018913 10.1023/a:1015062712767

[60] Cameron HL Perdue MH Stress impairs murine intestinal barrier function: improvement by glucagon-like peptide-2. J Pharmacol Exp Ther 2005;314:214–20 15798004 10.1124/jpet.105.085373

[61] Cameron HL Yang PC Perdue MH Glucagon-like peptide-2-enhanced barrier function reduces pathophysiology in a model of food allergy. Am J Physiol Gastrointest Liver Physiol 2003;284:G905–12 12736145 10.1152/ajpgi.00231.2002

[62] Benjamin MA McKay DM Yang PC Glucagon-like peptide-2 enhances intestinal epithelial barrier function of both transcellular and paracellular pathways in the mouse. Gut 2000;47:112–9 10861272 10.1136/gut.47.1.112 PMC1727982

[63] Cani PD Delzenne NM Gut microflora as a target for energy and metabolic homeostasis. Curr Opin Clin Nutr Metab Care 2007;10:729–34 18089955 10.1097/MCO.0b013e3282efdebb

[64] Bigorgne AE Bouchet-Delbos L Naveau S Obesity-induced lymphocyte hyperresponsiveness to chemokines: a new mechanism of fatty liver inflammation in obese mice. Gastroenterology 2008;134:1459–69 18471520 10.1053/j.gastro.2008.02.055

[65] Membrez M Blancher F Jaquet M Gut microbiota modulation with norfloxacin and ampicillin enhances glucose tolerance in mice. FASEB J 2008;22:2416–26 18326786 10.1096/fj.07-102723

[66] Yang R Han X Uchiyama T IL-6 is essential for development of gut barrier dysfunction after hemorrhagic shock and resuscitation in mice. Am J Physiol Gastrointest Liver Physiol 2003;285:G621–9 12773301 10.1152/ajpgi.00177.2003

[67] Adams RB Planchon SM Roche JK IFN-gamma modulation of epithelial barrier function. Time course, reversibility, and site of cytokine binding. J Immunol 1993;150:2356–63 8450217

[68] Coyne CB Vanhook MK Gambling TM Regulation of airway tight junctions by proinflammatory cytokines. Mol Biol Cell 2002;13:3218–34 12221127 10.1091/mbc.E02-03-0134 PMC124154

[69] Nusrat A Turner JR Madara JL Molecular physiology and pathophysiology of tight junctions. IV. Regulation of tight junctions by extracellular stimuli: nutrients, cytokines, and immune cells. Am J Physiol Gastrointest Liver Physiol 2000;279:G851–7 11052980 10.1152/ajpgi.2000.279.5.G851

[70] Sakaguchi S Furusawa S Oxidative stress and septic shock: metabolic aspects of oxygen-derived free radicals generated in the liver during endotoxemia. FEMS Immunol Med Microbiol 2006;47:167–77 16831203 10.1111/j.1574-695X.2006.00072.x

[71] Caplan MS Miller-Catchpole R Kaup S Bifidobacterial supplementation reduces the incidence of necrotizing enterocolitis in a neonatal rat model. Gastroenterology 1999;117:577–83 10464133 10.1016/s0016-5085(99)70450-6

[72] Ruseler-van Embden JG van Lieshout LM Gosselink MJ Inability of *Lactobacillus casei* strain GG, L. acidophilus, and *Bifidobacterium bifidum* to degrade intestinal mucus glycoproteins. Scand J Gastroenterol 1995;30:675–80 7481531 10.3109/00365529509096312

[73] Kleessen B Hartmann L Blaut M Fructans in the diet cause alterations of intestinal mucosal architecture, released mucins and mucosa-associated bifidobacteria in gnotobiotic rats. Br J Nutr 2003;89:597–606 12720580 10.1079/BJN2002827

[74] Bartholome AL Albin DM Baker DH Supplementation of total parenteral nutrition with butyrate acutely increases structural aspects of intestinal adaptation after an 80% jejunoileal resection in neonatal piglets. JPEN J Parenter Enteral Nutr 2004;28:210–22 15291402 10.1177/0148607104028004210

[75] Tappenden KA Drozdowski LA Thomson AB Short-chain fatty acid-supplemented total parenteral nutrition alters intestinal structure, glucose transporter 2 (GLUT2) mRNA and protein, and proglucagon mRNA abundance in normal rats. Am J Clin Nutr 1998;68:118–25 9665105 10.1093/ajcn/68.1.118

[76] Johnson-Henry KC Donato KA Shen-Tu G *Lactobacillus rhamnosus* strain GG prevents enterohemorrhagic *Escherichia coli* O157:H7-induced changes in epithelial barrier function. Infect Immun 2008;76:1340–8 18227169 10.1128/IAI.00778-07 PMC2292865

[77] Parassol N Freitas M Thoreux K *Lactobacillus casei* DN-114 001 inhibits the increase in paracellular permeability of enteropathogenic *Escherichia coli*-infected T84 cells. Res Microbiol 2005;156:256–62 15748992 10.1016/j.resmic.2004.09.013

[78] Martin GR Wallace LE Hartmann B Nutrient-stimulated GLP-2 release and crypt cell proliferation in experimental short bowel syndrome. Am J Physiol Gastrointest Liver Physiol 2005;288:G431–8 15388486 10.1152/ajpgi.00242.2004

[79] Thulesen J Hartmann B Kissow H Intestinal growth adaptation and glucagon-like peptide 2 in rats with ileal–jejunal transposition or small bowel resection. Dig Dis Sci 2001;46:379–88 11281189 10.1023/a:1005572832571

[80] Jeppesen PB Hartmann B Thulesen J Glucagon-like peptide 2 improves nutrient absorption and nutritional status in short-bowel patients with no colon. Gastroenterology 2001;120:806–15 11231933 10.1053/gast.2001.22555

[81] Chiba M Sanada Y Kawano S Glicentin inhibits internalization of enteric bacteria by cultured INT-407 enterocytes. Pediatr Surg Int 2007;23:551–4 17333210 10.1007/s00383-007-1895-9

[82] Zhang W Zhu W Zhang J Protective effects of glucagon-like peptide 2 on intestinal ischemia-reperfusion rats. Microsurgery 2008;28:285–90 18383347 10.1002/micr.20491

[83] Tsai CH Hill M Asa SL Intestinal growth-promoting properties of glucagon-like peptide-2 in mice. Am J Physiol 1997;273 1 Pt 1:E77–84 9252482 10.1152/ajpendo.1997.273.1.E77

[84] Dube PE Forse CL Bahrami J The essential role of insulin-like growth factor-1 in the intestinal tropic effects of glucagon-like peptide-2 in mice. Gastroenterology 2006;131:589–605 16890611 10.1053/j.gastro.2006.05.055

